# Cyclodextrins as Supramolecular Recognition Systems: Applications in the Fabrication of Electrochemical Sensors

**DOI:** 10.3390/ma14071668

**Published:** 2021-03-28

**Authors:** Bronach Healy, Tian Yu, Daniele C. da Silva Alves, Cynthia Okeke, Carmel B. Breslin

**Affiliations:** 1 Department of Chemistry, Maynooth University, Maynooth, Co. Kildare, Ireland; bronach.healy.2017@mumail.ie (B.H.); Tian.Yu.2020@mumail.ie (T.Y.); DANIELE.COSTADASILVAALVES.2021@MUMAIL.IE (D.C.d.S.A.); c22okeke@gmail.com (C.O.); 2 School of Chemistry and Food, Federal University of Rio Grande, Rio Grande 90040-060, Brazil

**Keywords:** cyclodextrins, inclusion complex, electrochemical sensors, biosensors, chiral recognition

## Abstract

Supramolecular chemistry, although focused mainly on noncovalent intermolecular and intramolecular interactions, which are considerably weaker than covalent interactions, can be employed to fabricate sensors with a remarkable affinity for a target analyte. In this review the development of cyclodextrin-based electrochemical sensors is described and discussed. Following a short introduction to the general properties of cyclodextrins and their ability to form inclusion complexes, the cyclodextrin-based sensors are introduced. This includes the combination of cyclodextrins with reduced graphene oxide, carbon nanotubes, conducting polymers, enzymes and aptamers, and electropolymerized cyclodextrin films. The applications of these materials as chiral recognition agents and biosensors and in the electrochemical detection of environmental contaminants, biomolecules and amino acids, drugs and flavonoids are reviewed and compared. Based on the papers reviewed, it is clear that cyclodextrins are promising molecular recognition agents in the creation of electrochemical sensors, chiral sensors, and biosensors. Moreover, they have been combined with a host of materials to enhance the detection of the target analytes. Nevertheless, challenges remain, including the development of more robust methods for the integration of cyclodextrins into the sensing unit.

## 1. Introduction

The development of sensors that can be employed in the selective and sensitive detection of an analyte has applications that extend from biomedical [1] to environmental analysis [2] and encompass wearable bio/chemical sensors [3,4] and sensors for monitoring urban air quality [5]. Electrochemical sensors can be designed for a variety of analytical applications with moderate cost and portability, combined with sensitive and selective detection. The fabrication of the sensor electrode, which acts as the transducer element, is one of the more significant and challenging steps. Fortunately, there is now a wide selection of new and novel materials with high electronic conductivity. The discovery of carbon nanotubes [6] and graphene [7], with their unique properties, has led to the emergence of alternative layered and nonlayered two dimensional (2D) materials that have become the focus of intense research [8]. Many of these materials have the capacity to give high sensitivity in electrochemical detection, while supramolecular systems can be integrated into the sensor design to give enhanced selectivity and sensitivity for the target analyte. Molecular recognition systems can vary from enzymes, antibodies, proteins [9], to aptamers (single-stranded DNA or RNA) [10], and to supramolecular molecules, such as cyclodextrins [11], calixarenes [12], cucurbiturils [13], crown ethers [14], and pillararenes [15]. 

Many of these systems have the ability to form host-guest complexes and cyclodextrins represent one of the most studied and well-known hosts.

**Figure 1:**
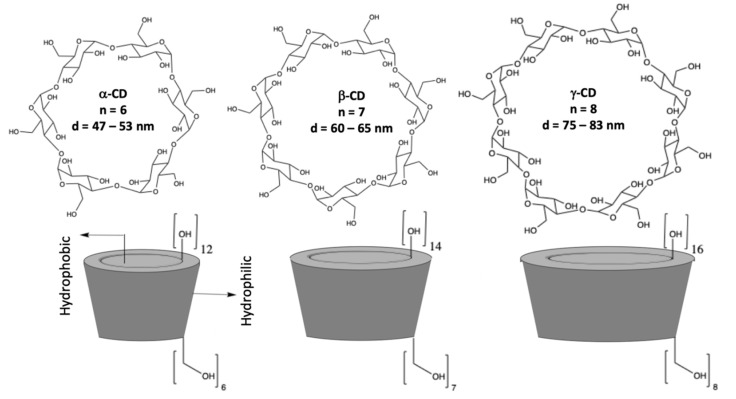
Structures of α-, β-, and γ-CDs (cyclodextrins) and a schematic of the conelike structures.

Cyclodextrins (CDs) are macrocyclic oligosaccharides consisting of α–1,4–linked D–glucopyranose units [16,17], as illustrated in Figure 1. They have a distinctive truncated cone structure, with the primary hydroxyl groups on the narrow side and the secondary hydroxyl groups on the wider base side of the cone. CDs differ in size with the more well-known and readily available having six, seven, and eight glucopyranose units, to give α-, β-, and γ-CDs, respectively. Cyclodextrins were first discovered in 1891 by Villiers and later developed by Schardinger [17]. Since then, there has been considerable interest in the applications of these systems, as they are readily available in high purity, are water soluble, biocompatible, and they can be functionalized using different synthetic methods and employed to give CD rotaxanes and pseudorotaxanes [18]. Moreover, they can incorporate a large variety of guest molecules (inclusion complexation) making them interesting in drug delivery [19], as adsorbent materials [20], and in the development of sensors, especially in the modification of electrodes to give highly selective electrochemical sensors [21,22].

**Figure 2:**
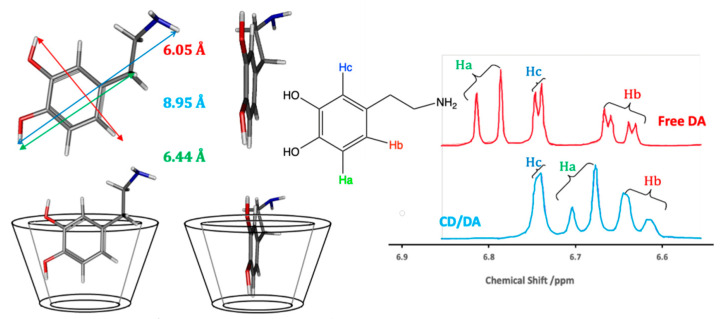
Schematic of the formation of an inclusion complex between β-CD and dopamine and the corresponding ^1^H NMR data, with an excess of the β-CD.

Although there is no single simple theory that can be used to describe the binding between the CD host and the guest molecule in solution, there is a general consensus that hydrophobic and van der Waals interactions are important in the formation of the inclusion complex [23,24,25]. In this case, the hydrophobic sites on the guest molecule are included within the hydrophobic cavity of the CD. Dipole-dipole, hydrogen bonding, and charge-transfer interactions have also been discussed for some guest molecules [25], illustrating the relevance of the properties of the guests, while the exclusion of cavity-bound high-energy water and conformational strain are also relevant factors [24]. Another important consideration is the size of the guest molecule, or the size of the hydrophobic component of the guest, compared to the internal diameter of the CD [23]. An illustration of this inclusion formation is given in Figure 2, where dopamine is considered as the guest molecule. The dimensions of dopamine indicate that it can form a host–guest complex with β-CD. The corresponding ^1^H NMR spectra of the aromatic region of dopamine show that not all the protons are affected in the same manner by the presence of the β-CD. There is a significant shift in Ha, a smaller shift in Hb and no observable change in Hc, suggesting that Hc remains outside the cavity.

A stable inclusion complex with a high binding constant, *K*, typically in the vicinity of 1 × 10^2^–1 × 10^4^ M^−1^, is only achieved if there is a good match between the size of the CD cavity and guest. This binding constant, which can also be described as a stability or complex formation constant, is defined in Equation (1) for the formation of a 1:1 inclusion complex. Here, G and H represent the guest and host, respectively, and C_HS_ indicates the host-guest complex. The equilibrium in the solution phase is represented in Equation (2). Some examples of typical binding constants are given in Table 1. These are usually obtained using analytical techniques, such as NMR or UV-visible spectroscopy, as the encapsulation of the guest molecule leads to alterations in its chemical and physical properties. These changes can be easily monitored, as illustrated in Figure 2, and normally involve titrations where the physiochemical properties of the guest molecule (or the host) are monitored as a function of its concentration.

(1) $$K=\frac{\left[C_{\mathrm{HG}}\right]}{\left[H\right]\left[G\right]}$$

(2) $$H+G⇋C_{\mathrm{HG}}$$

In this review, the applications of cyclodextrins in the development of electrochemical-based sensors and biosensors are described and discussed. There are a number of very good review articles already available describing the role of CDs in the development of colorimetric and/or fluorescence-based sensors [27,28], while Zhu et al. [29] have highlighted the advantages of employing CDs in electrochemical sensors. In this review, we consider the combination of CDs with a large variety of support/companion materials and describe the sensing performance of these CD hybrid materials in the detection of biomolecules, drug molecules, and environmental contaminants. In particular, we review the recent literature on combining CDs with graphene, carbon nanotubes, and other emerging two-dimensional materials. In addition, recent papers describing the integration of CDs into conducting polymers and the electropolymerization of CDs to give electrochemical sensors are introduced and discussed. Finally, the recent applications of CDs in the formation of biosensors and in chiral recognition are introduced.

## 2. The Role of Cyclodextrins in the Design of Electrochemical Sensors

**Figure 3:**
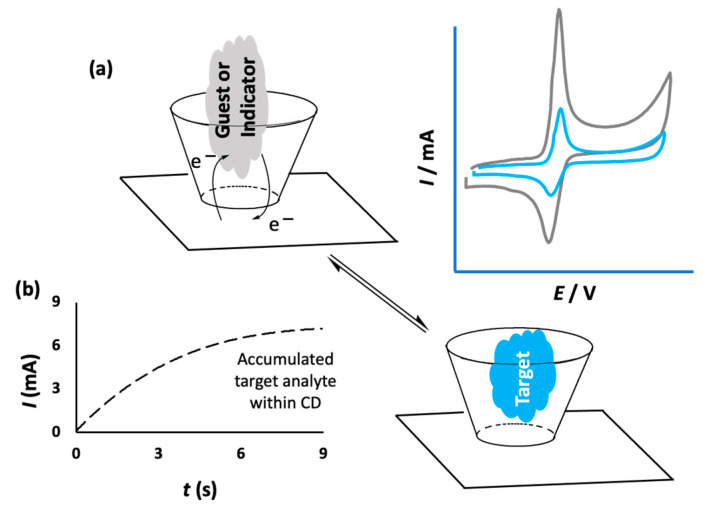
(**a**) Schematic of electron transfer for an included electroactive molecule and its exchange with a nonelectroactive target and the corresponding reduction in measured current; (**b**) the increasing current recorded following an accumulation period.

Cyclodextrins have been combined with a number of different materials with the aim of immobilizing them onto the surface of sensors. This approach gives rise to the formation of a CD array that has the potential to bind and trap the target analyte, facilitating selective and sensitive detection. The noncovalent binding between the immobilized CD and the analyte is sufficiently strong to capture the target analyte and confine it close to the electrode surface, but suitably weak to facilitate electron transfer and the oxidation or reduction of the analyte. This provides a signal, normally the flow of current, which can be related to the concentration of the analyte. Therefore, selective and sensitive detection can be achieved through the formation of an inclusion complex between the target analyte and CD, as illustrated in Figure 3a,b. In some cases, this can be facilitated by an accumulation period prior to the sensing event. This allows the target molecules to include and bind within the CD cavities [30,31]. As indicated in the schematic in Figure 3b, higher currents are obtained on increasing the accumulation period, and these accumulation times are typically no longer than a few seconds. Alternatively, competitive host–guest recognition, which is based on the competition between the target analyte and an indicator molecule for the same host, can be employed [32]. In this case, the indicator molecule is initially bound to the CD and when the target molecule diffuses to the electrode surface, the indicator molecule is displaced by the target, giving rise to a measurable change in the signal, as shown in Figure 3a. For example, Yang et al. used this approach with an immobilized CD in the electrochemical detection of cinchonine, an electrochemically inactive compound. The probe molecule, 1,4-hydroquinone (HQ), enters into the hydrophobic cavity of β-CD to form an inclusion complex and this gives rise to a current signal as the HQ is oxidized. However, when the HQ molecules are displaced by cinchonine, the current is lowered and this change in current can be used to quantify the concentration of cinchonine [33].

## 3. Cyclodextrins and Electrochemical Sensors

Cyclodextrins are well established in the field of supramolecular chemistry, but they are finding new applications by combining them with emerging materials to fabricate high performing electrochemical sensors. Different materials and approaches have been employed to immobilize the CD while retaining its supramolecular complexation properties and these are now introduced and discussed, highlighting the versatility of CDs in the design of electrochemical-based sensors.

### 3.1. Cyclodextrin Combined with Graphene

One of the more interesting combinations involve coupling the CDs with graphene and this approach is attracting considerable interest, particularly during the last decade. In 2010, it was first demonstrated by Guo et al. [34] that a synergistic effect was achieved when CDs were combined with graphene, giving rise to the impressive electrochemical detection of biomolecules and drugs. Graphene has a number of ideal characteristics and has been employed extensively in the development of electrochemical sensors [7,35]. These attractive properties include a high surface area, good conductivity, very good stability, and good thermal and mechanical properties, while it provides a metal free support for the CDs and it can be easily synthesized. While pristine graphene is difficult to form and maintain in this state, graphene oxide (GO) can be formed readily using the well-known modified Hummers method [36,37,38]. This involves the oxidation of graphite and as the oxidation proceeds the interlayer spacing increases and exfoliation can be achieved through ultrasonication to give GO sheets. These sheets can then be reduced chemically [39] or electrochemically [40,41] to give the more conducting reduced GO (rGO). The rGO, with its good conductivity and efficient electron transfer, is more suitable in the preparation of electrochemical-based sensors. The complete reduction of GO to rGO is difficult to achieve and the resulting rGO will inevitably contain oxygen-containing functional groups. However, these functional groups can be employed to covalently attach CDs to the graphene sheets [42]. Hydrogen bonding between the –OH groups on the CDs and the oxygen-containing groups on GO and rGO favor the attachment of the CDs onto the GO/rGO sheets [21,43]. Indeed, it was shown by Guo et al. [34] that the O–H stretching vibration shifted from 3700 cm^−1^ for free OH to 3429 cm^−1^ for the OH groups in the CD/rGO composite. This large red shift was attributed to the formation of strong hydrogen bonds between the CDs and the oxygen-containing groups on the rGO. Moreover, it has been shown, using surface analytical measurements, such as SEM and TEM, that the CD modified rGO sheets are less prone to aggregation, indicating that the incorporated CDs minimize the restacking effects normally seen with rGO, maintaining the favorable high surface areas associated with the well-dispersed rGO sheets [44].

**Figure 4:**
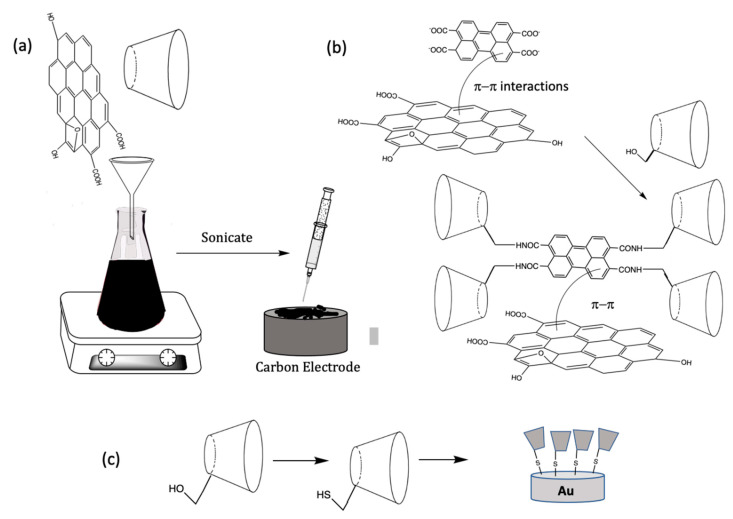
Schematic of (**a**) the solution-based fabrication of a CD/rGO sensor; (**b**) noncovalent π–π interactions with rGO coupled with a functionalized CD and (**c**) covalent attachment.

The noncovalent methodologies used to form the rGO/CD-based sensors are generally very simple and can be carried out with no significant chemical modifications. Typically, GO is dispersed in solution and then mixed with the CDs, to form stable aqueous dispersions, and drop cast onto a carbon-based electrode, such as glassy carbon [34], screen printed electrodes [45], or fabricated as carbon paste electrodes [46,47,48]. The GO/CD can then be reduced chemically using reducing agents, such as hydrazine [44,45], or the more environmentally acceptable ascorbic acid [49] to give rGO/CD. Alternatively, the GO/CD can be electrochemically reduced by cycling the applied potential to values in the vicinity of −1.4 V vs. SCE (saturated calomel electrode) [21]. In some studies, the GO is reduced initially to form rGO, which is then combined with the CD [50], while the CD has also been immobilized onto the rGO modified glassy carbon electrode by simple immersion in the CD solution for about 5 h [51]. These simple methodologies are illustrated and summarized in the schematic provided in Figure 4a.

In addition to the simple strategies illustrated in Figure 4a, a number of other modifications have been explored and these include the covalent attachment of the CD or a functionalized CD to graphene, functionalized CDs incorporated through noncovalent attachments, the incorporation of polymeric reagents and a variety of metal nanoparticles. Various functionalized CDs have been employed to generate CD/rGO sensors [52,53]. Although this is a time-consuming approach compared to the simple wet chemical methods initially discovered, functionalized CDs can be used to covalently link the CD to the graphene nanosheets, giving rise to stronger binding and a free dangling attached CD that is more accessible to the target analyte. Functionalized CDs can also alter the formation of the inclusion complex and therefore the functionalized groups can be selected to tailor the binding between the CD and the target analyte, to give improved sensitive and selective detection. Furthermore, aromatic functional groups can be utilized to form dense self-assembled monolayers using noncovalent π–π interactions [54]. These approaches are illustrated in the schematic presented in Figure 4b. For example, HP–β-CD (2–Hydroxylpropyl–β-CD) decorated rGO nanosheets were formed with the assistance of microwave irradiation. In this analysis, the CD macromolecule was covalently grafted onto the surface of the rGO nanosheets through the formation of ester bonds [55]. Other functionalized CDs have been employed and these include 2,6–dimethyl–β-CD [56], mercapto–β-CD [57], and thio–β-CD (SH–β-cyclodextrin) [58,59,60,61,62], with a schematic of the thiolated system shown in Figure 4c. In addition, dopamine–β-CD [63] and pyrene–β-cyclodextrin [64] have been immobilized onto rGO using noncovalent π–π interactions to give efficient electrochemical sensors.

Polymeric materials and other linker molecules have also been employed to coat, modify, or functionalize the rGO sheets, and these include poly(diallyl dimethyl ammonium chloride) [44] and 3,4,9,10–perylene tetracarboxylic acid, which act as a bridge between the rGO and CD [65]. Prussian blue–chitosan [66] and cytochrome c [67] have also been combined with rGO and CDs and used to improve the electron transfer kinetics. Nafion, a negatively charged perfluorsulfonated ion-exchange polymer, which is normally employed to repel anionic analytes, has been combined with rGO and CD [68]. Likewise, the functional groups on chitosan (protonated amine groups, $-{\mathrm{NH}}_{3}^{+}$), a well-known biopolymer, have been exploited to attract negatively charged molecules [69].

Studies have also focused on combining metal nanoparticles with the CD and rGO to give highly efficient electron transfer. This is not surprising as metal nanoparticles have excellent conductivity, very good electrocatalytic activity and in many cases have good biocompatibility, while they are more cost effective than their bulk counterparts and have been employed extensively in the electrochemical detection of various molecules [70,71]. More recently, these nanoparticles have been combined with GO/rGO and CDs to further enhance the rate of the electron transfer reaction. For example, a simple one-pot synthesis was employed to give the in situ reduction of HAuCl_4_ to yield gold nanoparticle decorated rGO with β-CD [72]. It was shown that the β-CD not only acted as a dispersant but also served as a reducing agent, facilitating the reduction of GO to rGO and the conversion of ${\mathrm{AuCl}}_{4}^{-}$ to gold nanoparticles.

### 3.2. Cyclodextrins Combined with Carbon Nanotubes

Another interesting combination involves the blending of carbon nanotubes (CNTs) with CDs. CNTs are also well known for their high electronic conductivity, surface areas, and very good stability, and have been employed in the fabrication of a number of sensors [70,71,73]. CNTs can be formed as single-walled (SWCNT) or multiwalled nanotubes (MWCNT), distinguished by the number or graphitic layers folded over to form the tubes. In the last decade there has been considerable interest in modifying CNTs with CDs and there is now ample evidence to show the beneficial effects of this combination. Recent studies have demonstrated enhanced electron-transfer reactions at these composites, combining the attractive properties of the CNTs with the supramolecular inclusion complexation characteristics of the CDs [74,75].

The CNTs can be easily combined with the CDs through a simple wet chemical synthesis, similar to that used with the graphene sheets. This is aided by the good dispersion of the CNTs in a CD solution. Normally, for efficient dispersion, the CNTs are treated in nitric acid to generate –COOH groups [76]. Although this is an efficient strategy, the generation of –COOH groups enhances the dispersion of the CNTs, leading to environmental concerns as these functionalized CNTs are readily released into the aquatic environment [77]. In contrast, unmodified CNTs and CDs can be sufficiently well dispersed with good stability in aqueous or polar media [30,78,79]. This is normally explained in terms of the van der Waals forces between the MWCNTs and the CDs, where the CDs are sandwiched between the MWCNTs [80]. This good dispersion also facilitates the formation of a homogenous modified electrode. Indeed, it was shown by Alam et al. [78] that a glassy carbon electrode modified with MWCNTs gave rise to a heterogeneous coverage, while a uniform morphology was observed with the MWCNT/βCD. This was attributed to the hydrophilic properties of the CDs, enabling good dispersion in water.

**Figure 5:**
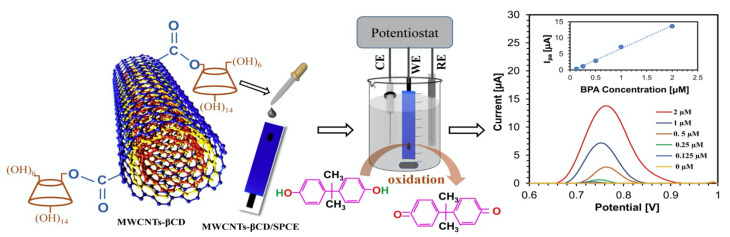
CD-linked multiwalled carbon nanotubes (MWCNTs-βCD) deposited onto screen printed electrodes (SPCE) and employed in the detection of Bisphenol A. Reproduced with permission from Ali et al. [92], Sens. Actuators B Chem.; published by Elsevier, 2020.

Alternatively, the CDs can be grafted onto the CNTs functionalized with –COOH groups. This generally involves a two-step process, where the CNTs are firstly functionalized with –COOH groups and then the CDs are covalently linked through the –COOH groups. This approach is highlighted in Figure 5. This normally gives rise to about 14% of the available –COOH groups linked to the CDs [81]. Disulfide bridged β-CD dimer-functionalized MWCNTs have also been synthesized and employed to give enhanced conductivity and efficient electron transfer [82].

In a similar manner to that employed with the graphene-based system, the CNTs and CDs have been combined with and without other additives and fabricated as carbon paste electrodes [11,31] and drop-cast onto glassy carbon or screen printed electrodes. Likewise, the CNTs have been combined with functionalized CDs [83,84] and the CNTs/CDs have been further modified with metal nanoparticles, including gold [85,86,87], copper [88], cobalt [89], silver [90], and platinum [91].

### 3.3. Cyclodextrins Combined with Conducting Polymers

Cyclodextrins have been immobilized successfully within a number of polymer matrices and used as electrochemical sensors. Some of the better studied polymers come from the family of conducting polymers and include polypyrrole (PPy), polyaniline (PANI), and polythiophene derivatives, such as poly(3,4–ethylenedioxythiophene) (PEDOT). These polymeric materials are interesting as they have a high surface area, a highly porous surface, where the porosity can be further controlled by varying the rate of electropolymerization, have ion exchange properties, and provide a conducting matrix. These polymers are easily formed at a substrate electrode through the electropolymerization of the corresponding monomers in aqueous solutions. For example, PPy can be formed at a variety of conducting substrates through the electropolymerization of pyrrole in the presence of a dopant at potentials in the vicinity of 0.6 to 0.8 V vs. SCE [93,94] to generate an adherent conducting film.

Cyclodextrins have been incorporated within a number of conducting polymers, and it has been shown that the CDs play a significant role in the detection of the target molecules, with the CD immobilized films outperforming the other combinations. This clearly illustrates that the immobilized CDs retain their supramolecular complexation properties. For example, PANI films have been combined with β-CD and –COOH functionalized MWCNTs and used in the selective detection of dopamine [95]. The MWCNTs promoted electron transfer, while a supramolecular complex was formed between β-CD and dopamine. The sensing performance of the β-CD-MWNTs/PANI film was superior to the MWCNTs/PANI, PANI, or substrate glassy carbon electrodes. This combination of MWCNTs, PANI, and β-CD has been employed in the fabrication of sensors for the determination of phenylalanine [96], dopamine [97], ciprofloxacin [98], cocaine [99], and fungicides and herbicides, such as pyrimethanil [100], chlorophenoxy compounds [101,102], and bentazone [103].

Substituted aniline monomers, such as N–acetylaniline, have also been electropolymerized in the presence of β-CD and employed as electrochemical sensors for the detection of guanine and adenine [104] and in the simultaneous detection of levodopa and carbidopa in pharmaceutical formulations [105]. MWCNTs have been added to further enhance the kinetics of the electron transfer reaction and these ternary PAcANI/β-CD/CNT hybrids have been used in the simultaneous detection of serotonin and dopamine [31], and for the quantification of hydroquinone [106], while the good conducting properties of graphene have been exploited to give efficient PAcANI/β-CD/rGO electrochemical sensors [33,107].

A number of CD-modified polypyrrole films have been fabricated and employed as sensors. These polypyrrole/β-cyclodextrin films (PPy/β-CD) can be formed by the simple electropolymerization of pyrrole in the presence of the β-CD and a supporting electrolyte to give PPy/β-CD adherent films. These polymeric materials have been employed in the detection of catecholamine compounds [108], various neurotransmitters [109,110,111], and methyl paraben [112], and utilized as an impedimetric sensor to detect rocuronium bromide, which is a muscle relaxant [113]. By employing anionic β-CDs, such as sulfonated or sulfated CDs (sβ-CD), the anionic β-CDs can be incorporated as dopants within the PPy matrix. As these are large and immobile dopants, they are not expelled as the polymer is reduced and are very well immobilized. These PPy/sβ-CD films have been employed in the electrochemical detection of dopamine [114,115], urea [116,117], and viologens [118]. Similar to the strategy employed with the PANI system, MWCNTs [119,120] and rGO [121,122,123] have been added to the PPy/CD polymers to enhance conductivity and the performance of the sensors.

PEDOT, which can be prepared by the chemical or electrochemical oxidation of EDOT, has also been combined with β-CD to develop electrochemical sensors. For example, a nanostructured PEDOT/β-CD was prepared by oxidizing EDOT with FeCl_3_ in the presence of β-CD. Again, it was shown that the PEDOT/β-CD outperformed the PEDOT in the sensitive and selective sensing of hyperoside and shikonin [124]. PEDOT/β-CD films have also been utilized in the electrochemical quantification of acetaminophen (paracetamol) [125], creatinine [126], sulfur oxoanions, and nitrite [127]. PEDOT and sulfated β-cyclodextrin (PEDOT/sβ-CD) films deposited onto gold were investigated for the simultaneous detection of ascorbic acid and catecholamines, giving good simultaneous analysis [128].

### 3.4. Electropolymerization of Cyclodextrins

An interesting alternative to the physically immobilized CD and the covalently linked CD supramolecules is the electropolymerization of the CDs to generate an adherent insoluble CD polymer. While the physically adsorbed CD supramolecules, which are soluble, tend to dissolve from the surface, leading to a progressive loss of the CDs from the surface, the insoluble CD polymer is retained. Moreover, it has been shown in several studies that this polymer has good conducting properties [129], making it an ideal candidate for the development of electrochemical-based sensors. Furthermore, it can be combined with a range of other conducting materials, such as CNTs [130,131] and rGO [129,132].

The CD polymers, poly-β-CDs, are normally formed using cyclic voltammetry where the substrate electrodes are cycled between the potential limits of −2.0 V and 2.0 V to 2.3 V vs. SCE [133], or with a smaller electrochemical window between approximately −0.8 to 1.3 V vs. SCE. Acidic conditions are generally used with the polymer being generated in slightly acidic phosphate buffer [133,134], or in the presence of HClO_4_ [135,136]. These poly-β-CDs have been deposited at carbon paste electrodes [136,137], glassy carbon [138,139,140], and rGO decorated screen printed electrodes [129], and combined with CNTs [141,142], rGO [132,133,143], boron-doped rGO [134], TiO_2_ [144], and gold nanoparticles [145].

**Figure 6:**
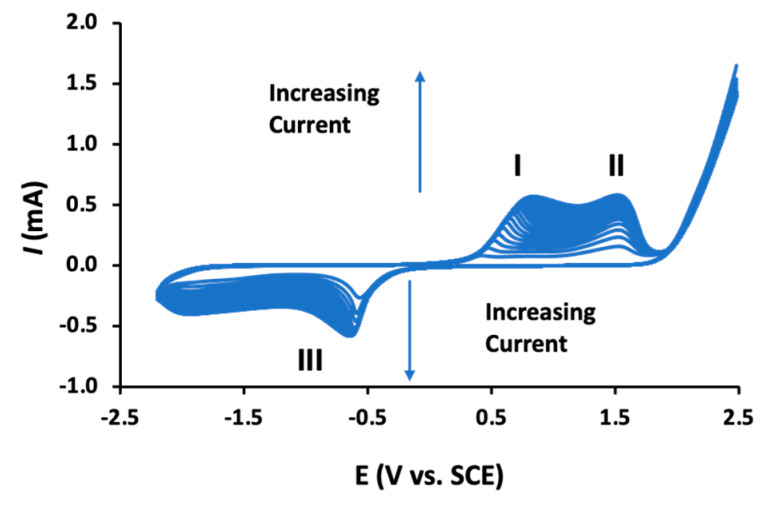
Cyclic voltammograms recorded during the electropolymerization of β-CD in an acidified phosphate buffer, I and II refer to oxidation waves and III to a reduction wave.

Despite the growing number of publications where these poly-β-CDs are being used in the design of electrochemical sensors, there is relatively little known about the electropolymerization mechanism. As the polymeric material is formed, the voltammograms change with the emergence of well-defined redox peaks, as illustrated in Figure 6. Clear oxidation waves (peaks I and II) are observed and a well resolved reduction wave (peak III) is evident at about −0.6 V vs. SCE. These peaks increase with increasing cycling until about 15–20 cycles are reached, at which point the currents become essentially constant, indicating no further or little growth of the polymer. The oxidation waves (peaks I and II) have been attributed to the oxidation of the β-CD supramolecules. Indeed, β-CDs have been employed as reducing agents, clearly indicating that these β-CDs can be oxidized [72]. The electropolymerization mechanism has been described as a radical polymerization reaction, where a radical cation is formed facilitating the formation of dimers, trimers, and eventually the dark-colored poly-β-CD. It has been suggested that it is the –CH_2_–OH of the β-CD which is oxidized, as the C–1 position is blocked by the glycosidic linkage [136]. This active site can form a dimer with an adjacent β-CD molecule but also form a covalent bond with the carbon substrate electrode [139], which is consistent with the good adherence of the polymer to the substrate electrode. It is also well established that the primary alcohol groups are susceptible to oxidation, giving rise to the formation of aldehyde and/or a carboxylic acid group [146]. These carboxylic acid groups can react with the primary hydroxyl groups through an esterification reaction to give the β-CD dimer [139]. As β-CD dimers, trimers, and higher polymeric units are formed, the solubility in aqueous media will decease significantly in the diffusion layer, leading to the precipitation of the insoluble poly-β-CD.

Although the electropolymerization mechanism is not fully understood and the origins of peaks I, II, and III in Figure 6 have yet to be fully explained, these poly-β-CD-based sensors have been employed successfully. Indeed, these sensors have been used in the electrochemical detection of biomolecules [129,131,135,142,145], insecticides [132,140,141], and heavy metal ions [136].

### 3.5. Other Conducting Materials Combined with Cyclodextrins

In addition to the materials described above, a number of other promising combinations comprising CDs as the molecular recognition agent have been explored in the development of sensors. Some of the more interesting materials include highly porous materials, such as ordered mesoporous silicas and mesoporous carbon, and 2D layered materials with good conducting properties and high surface areas, such as graphitic carbon nitride, MXenes, layered double hydroxides (LDHs), and 2D layered TiO_2_ nanosheets, which have good hydrophilic character. Additional new and emerging materials include carbon nanohorns (CNHs), which are carbon nanomaterials with a similar shape to CNTs but with horn-shaped sheaths composed of graphene sheets. In terms of ordered mesoporous silica, these materials possess an ordered porous structure, with uniform pore sizes and a high surface area. SBA-15, a hexagonal mesoporous silica, has been functionalized with CDs to form sensors for the determination of nitrophenol isomers [147], while mesoporous carbon has been combined with CDs and gold nanoparticles for the detection of p-nitrophenol [148].

Graphitic carbon nitride has a layered 2D structure, with the graphitic planes connected by amino groups, with good conductivity and is normally formed through the pyrolysis of melamine. This layered material has been combined with graphite, mineral oil, and β-CD to form a carbon paste electrode and employed in the electrochemical detection of linagliptin, a hypoglycemic drug molecule [149]. A carboxymethyl–β-cyclodextrin, serving as the polyanion, was layered with Mg–Al LDH and deposited onto a glassy carbon electrode to give the capacitive detection of acetaminophen. The LDH nanosheets provided a rigid inorganic matrix, while supramolecular recognition was achieved with the CD [150]. Fu et al. decorated 2D TiO_2_ nanosheets with gold nanoparticles, to enhance conductivity and provide a gold surface to facilitate the formation of Au–S bonds with a mono–6–thio–β-CD [151].

**Figure 7:**
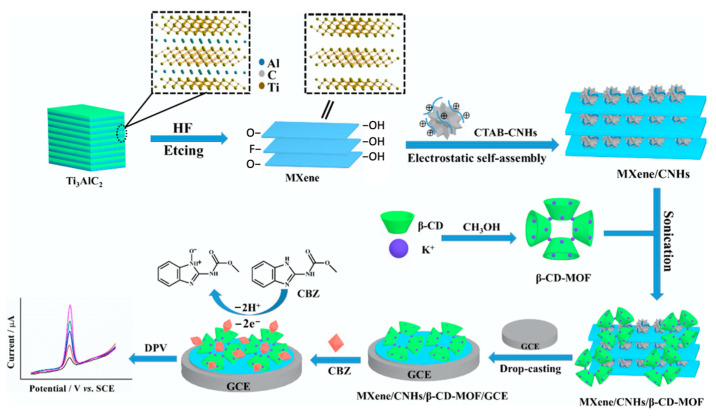
Synthetic route of the MXene/CNHs/β-CD-MOFs and the sensing strategy for carbendazim. Bulk Ti_3_AlC_2_ is etched and exfoliated, then combined with carbon nanohorns (CNHs), decorated with β-CD-MOF and drop casted onto a glassy carbon electrode (GCE). Reproduced with permission from Tu et al. [154], J. Hazard. Mater.; published by Elsevier, 2020.

The CNHs have been combined with β-CD to give a hybrid material with extremely high electrochemiluminescent (ECL) activity for luminol and an effective and selective ECL biosensor [152], while Kingsford et al. formed an aqueous dispersion of CHNs and β-CD using a simple ultrasonication process and then drop-cast the dispersion onto a glassy carbon electrode to produce an electrochemical sensor [153]. In a more recent study, MXenes, CNHs, β-cyclodextrin, and metal–organic frameworks were combined and exploited for the electrochemical detection of carbendazim [154]. This strategy is summarized in Figure 7. The MXene nanosheets are initially formed through dissolution of the Al layer in the bulk Ti_3_AlC_2_ and then combined with CNHs, CD-MOF, and drop-cast onto a glassy carbon electrode.

CDs have also been combined with various nanoparticles/nanostructures by simply mixing (with sonication) and then drop-casting the slurry onto a glassy carbon substrate. This strategy has been employed with SrTiO_3_, a perovskite with good electronic conductivity and chemical inertness, to give SrTiO_3_/β-CD and employed in the electrochemical detection of As(III) [155]. Similarly, various metal nanoparticles have been combined with CDs to form decorated glassy carbon electrodes. For example, Daneshvar and Rounaghi [156] employed trimetallic Au/Ag/Pd nanoparticles with β-CD for the detection of carbamazepine. Similarly, a number of carbon paste electrodes modified with metal nanoparticles and β-CD have been formed and utilized as electrochemical sensors and this includes gold [157,158] and platinum nanoparticles [46], while gold substrate electrodes have been modified with gold nanoparticles, β-CD, and Nafion [159].

### 3.6. Cyclodextrin-Based Biosensors

**Figure 8:**
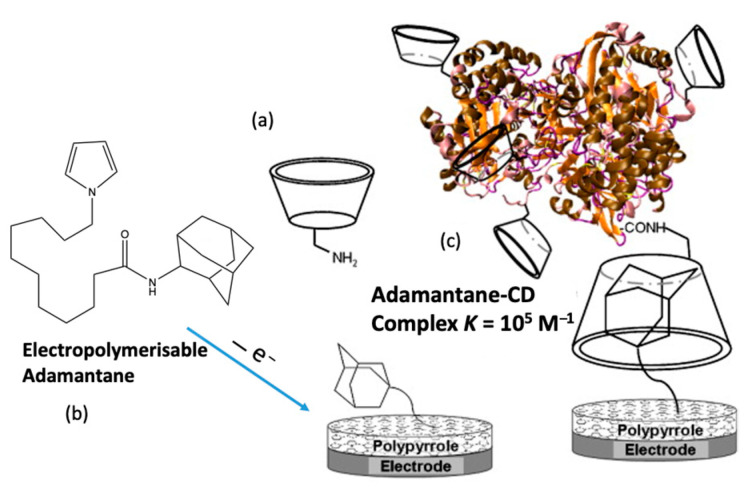
Supramolecular assembly of β-CD tagged glucose oxidase (GOX) and an adamantine-modified electrode: (**a**) representation of mono-6-amino-β -CD, (**b**) electrogeneration of poly(adamantane-pyrrole), and (**c**) polymerized adamantane/β-CD complex. Reproduced with permission from Cosnier and coworkers [119], Biosens. Bioelectron.; published by Elsevier, 2009.

It is well established that electrochemical-based biosensors can be constructed using molecular recognition elements, such as proteins, enzymes, and more recently aptamers. However, there is also clear evidence that the addition of cyclodextrins can lead to a further improvement in the sensitivity and selectivity of biosensors. This supramolecular-centered approach can be employed to increase the loading of the enzyme/protein. Moreover, the CD has a hydrophobic cavity, providing an ideal microenvironment for the enzyme. Among the extensive range of host–guest complexes, the adamantane–β-CD inclusion complex [160] is especially useful in the design of electrochemical-based biosensors [161,162]. The CD adamantane host–guest complex serves to dock the biological recognition element of the sensor, facilitating its recognition of the target analyte. Coupling is normally achieved with adamantane carboxylic acid [163,164], but in addition, adamantane derivatives can be used. For example, an adamantane-pyrrole derivative has been employed as an affinity binding polymer [119] and pyrene-adamantane was used to form a biosensor for the detection of glucose [165]. This strategy is summarized in Figure 8, where pyrrole is coupled to adamantane, facilitating the deposition of a polypyrrole film with free adamantane that can form an inclusion complex with the CD. This in turn captures the β-CD tagged glucose oxidase (GOX), anchoring it to the electrode surface.

Thiol groups serve as very good linkages enabling the attachment of CDs and polythiolated CDs to the biosensor surface [166]. If the entire surface is covered, this hydrophilic surface layer (due to the hydrophilic exterior of the CD) reduces considerably the nonspecific interactions, and this is especially important in the development of immunosensors. In addition, the CD can be polymerized using the well-known method that uses strong alkaline conditions coupled with epichlorhydrin to give a polymer with a β-CD content of about 52% [167], or indeed the CD can be electropolymerized to give a polymer layer or electropolymerized in the presence of enzymes to give an enzyme immobilized polymer layer [130].

**Figure 9:**
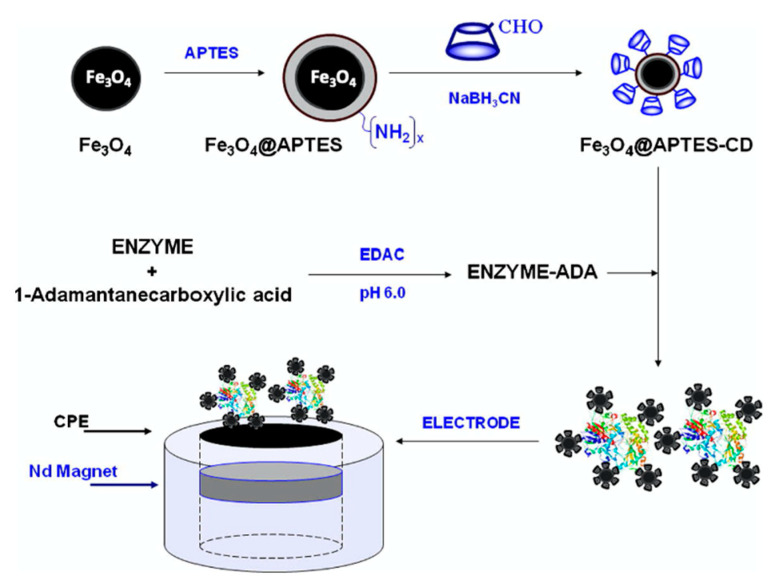
Illustration of the formation of magnetically anchored enzyme using CD-coated superparamagnetic Fe_3_O_4_ nanoparticles. Reproduced with permission from Diez et al. [161], J. Colloid Interface Sci.; published by Elsevier, 2012.

Other additives have been combined with the CDs, and graphene is a good option, as it provides good conductivity with high surface areas. This combined with the CD host-guest interactions gives rise to high loadings of the biological recognition agent. This approach has been utilized in the development of an immunosensor for the detection of carcino-embryonic antigen (CEA), an important biological marker of colorectal adenocarcinoma [168]. Likewise, carbon nanotubes have been integrated into the supramolecular biosensors due to their high electrocatalytic properties and employed in the detection of organophosphates [169] and glucose [119]. Moreover, gold [170], platinum [171,172], and silver [173] nanoparticles have been added to enhance the rate of the electron-transfer reaction. Likewise, enzyme-loaded nanoparticles have been magnetically immobilized in the fabrication of biosensors for xanthine and catechol detection [161] and this strategy is illustrated in Figure 9. The magnetic nanoparticles are functionalized with amine groups and then coupled with the CDs through the reaction of the NH_2_ and CHO groups, combined with the enzyme and cast onto a magnetic substrate.

Another important element in the design of biosensors are mediators which are frequently utilized, and ferrocene and its derivatives are very effective as mediators [174]. However, their leaching from the sensor remains challenging, affecting the overall stability of the biosensor. Ferrocene forms a strong inclusion complex with β-CD [174] and this has been exploited to improve the stability of the ferrocene-based mediators. This ferrocene/β-CD combination has been used in the amperometric detection of glucose [175,176] and as an electrochemiluminescence biosensing platform for Hg(II) [177].

The ferrocene/β-CD inclusion complex has also been applied in the assembly of aptamer-based sensors [178,179,180]. Aptamers are single-stranded RNA or DNA oligonucleotides and they can bind with high affinity and specificity to their target molecules. They can be engineered with different sequences to recognize a variety of target molecules. Compared with receptor proteins or antibodies, aptamers have good thermal stability and are cost-effective, making them widely applicable in the design of biosensors. Upon binding to their target molecules, the aptamers fold their flexible, single-stranded chains into a three-dimensional (3D) structure, moving either closer or further away from the surface. Provided a conducting pathway exists, these interactions can be converted into an electrochemical signal. This strategy was used by Wu et al. [179] and Xue et al. [178] to form electrochemical aptasensors for the detection of tetracycline and thrombin, respectively. In both cases, a thiolated β-CD was attached to a gold substrate and the aptamer was labeled with ferrocene, enabling the formation of an inclusion complex between the ferrocene and β-CD, while anchoring the aptamer to the surface. On recognizing the target analyte, the aptamer changes its configurations moving the ferrocene/aptamer/target complex away from the surface and reducing the electrochemical signal [179], or closer to the surface to increase the signal [178].

### 3.7. Enantioselective Cyclodextrin-Based Electrochemical Sensors

Enantioselective sensors or chiral-based recognition sensors are centered on the principle that the sensor can preferentially recognize one of the enantiomers of a chiral molecule. This is especially relevant to drug molecules as the enantiomers can exhibit very different therapeutic effects, with one of the enantiomers giving the desired response, but the other having serious side effects. In recent years, β-CD has been recognized as a chiral selector and employed as the chiral element in electrochemical-based sensors. A variety of stereoisomer molecules, including tryptophan [181,182,183,184], phenylalanine [65,185], dihydroxyphenylalanine (DOPA) [186,187], cystine [188], moxifloxacin hydrochloride [189], and clopidogrel [46] have been detected with the β-CD, showing a high preference for one of the enantiomers.

**Figure 10:**
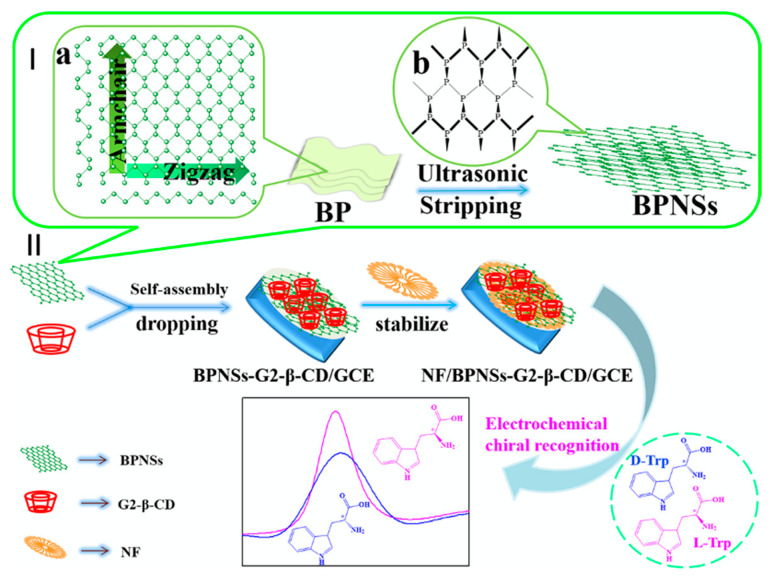
Schematic representation of the liquid-phase exfoliation of (**a**) black phosphorus (BP) to give (**b**) nanosheets of black phosphorus (BPNSs), shown in **I**, and fabrication of a Nafion (NF) BPNSs/β-CD modified electrode and its application in the electrochemical recognition of tryptophan enantiomers shown in **II**. Reproduced with permission from Zou and Yu [184], Mater. Sci. Eng. C; published by Elsevier, 2020.

The chiral recognition property of β-CD is based on its ability to form stronger inclusion complexes with one enantiomer. For example, β-CD forms a stronger inclusion complex with L-tryptophan compared to D-tryptophan [181,184], as stronger hydrogen bonds can be formed between the amino group on L-tryptophan and the secondary hydroxyl on the β-CD rim, due to the orientation of the included amino acid [183]. Likewise, β-CD shows a preference for L-phenylalanine, and this has been attributed to more facile hydrogen bonding between the amino group of L-phenylalanine and the secondary OH groups on the rim/edge of the β-CD [65]. The degree of enantioselective recognition can be determined by changes in the peak currents arising from the oxidation of the two enantiomers, while shifts in the peak potential, Δ*E*p, are also a good indication of chiral recognition in electrochemical-based sensors [189]. For example, typical changes in the peak currents are illustrated in Figure 10, where nanosheets of black phosphorus and β-CD are used to discriminate between the enantiomers of tryptophan, with the L-tryptophan being preferentially detected.

For analytes that have poor electroactivity, or are not electroactive, ferrocene can be employed. This strategy is based on the strong inclusion complex that forms between β-CD and ferrocene. As long as the ferrocene is maintained within the cavity, high currents are measured. However, once ferrocene is replaced by the preferred enantiomer, the current decreases and this drop in the peak current arising from the electroactive ferrocene can be used to determine the degree of the enantioselective recognition [65].

While there is normally good agreement in terms of the preferred enantiomer included within the CD cavity, there are also some conflicting results. For example, Chen et al. [186] concluded that the β-CD exhibited stronger enantioselectivity towards D-DOPA, while Ates et al. [187] observed poor discrimination of D- and L-DOPA using β-CD, but favorable interactions between L-DOPA and γ-CD. This may indicate that the other components of the sensor are also important. Indeed, Upadhyay et al. [189] observed synergistic effects between graphene and β-CD in the discrimination of the enantiomers of moxifloxacin.

## 4. A Comparison of the Performance of the Cyclodextrin Modified Electrodes as Sensors

It is clearly evident from the previous sections that the integration of CDs, with their supramolecular interactions, into the sensor assembly can be employed in the detection of several molecules. A summary and comparison of some of the cyclodextrin-based sensors are shown in Table 2, Table 3, Table 4 and Table 5. The molecules are divided between environmental contaminants (Table 2), essential biomolecules focused on neurotransmitters and amino acids (Table 3), and biosensors (Table 4). Lastly, the performances of drug molecules and flavonoid sensors are illustrated in Table 5. It is clearly evident that a large number of molecules have been detected using a variety of CD-based sensors. The CDs, mainly β-CD, have generally been combined with conducting materials, including graphene sheets/reduced graphene, CNTs, (normally MWCNTs), and a range of metal nanoparticles. The performance of the sensors can be compared using the limit of detection (LOD), which is defined in Equation (3), where *σ*_b_ is the standard deviation in the baseline and *m* represents the sensitivity of the sensor and corresponds to the slope of the linear calibration plot. In some cases, the standard deviation is found using the lower concentrations of the analyte. These computed LOD values can often overestimate the sensing ability of a sensor, and therefore, the linear region can be more useful in assessing and comparing the performances of sensors. For example, the LOD value was computed as 6.64 pM, while the linear region only extended to a lower concentration of 0.04 μM in the electrochemical detection of methocarbamol [190].

(3) $$LOD=\frac{3\sigma_{b}}{m}$$

In terms of the environmental contaminants, Table 2, it is no surprise that highly carcinogenic compounds, such as bisphenol A, nitro-and chlorophenols, and a number of herbicides and pesticides have been selected as the target analytes. Many of these compounds have aromatic rings and this will facilitate the formation of an inclusion complex between the CD and the contaminant. These aromatic rings can include within the hydrophobic cavity of the CD and this inclusion complex allows the accumulation of the molecules at the surface, giving rise to enhanced electrochemical detection.

The interactions between metal ions, such as heavy metal ions, and CDs are largely unexplored, and this may be connected to the difficulties involved in obtaining good quality crystalline materials for structural analysis. However, the secondary hydroxyl groups of the CDs can deprotonate and form complexes with metal ions, but this usually leads to the generation of a sandwich-type complex where the CD molecules become connected, through the metal ions [191]. This is unlikely to occur with the immobilized CDs, and therefore it appears that the good performance seen in the electrochemical stripping of the metal ions (Table 2) is largely due to the graphene sheets. However, the CD plays a role. There is clear evidence to show that the CDs can prevent the restacking of the graphene sheets through the formation of hydrogen bonds with GO [21,34], and this will enhance the performance of the sensors. These synergistic interactions between CDs and graphene have led to the development of several rGO/CD-based sensors and this is clearly seen in Table 2, Table 3, Table 4 and Table 5. Likewise, CNTs can be well dispersed in CD solutions and a number of sensors have been developed using MWCNTs combined with CDs.

Similarly, for the drug molecules, flavonoids, amino acids, and biomolecules, rGO is frequently combined with CDs (Table 3 and Table 5). Again, many of the systems contain hydrophobic aromatic ring structures and these can include within the hydrophobic CD cavity, to give an inclusion complex. This gives rise to impressive detection with linear regions, extending over a wide concentration range, covering the therapeutic levels for many drugs, while also being able to detect concentrations at the nM levels. This ability to detect concentrations at the nM level can be employed not only in biological samples, but also in the analysis of antibiotics and/or drug molecules in wastewater, where concentrations of nM and lower exist.

In terms of the essential biomolecules, dopamine, ascorbic acid, and uric acid coexist in biological systems and changes in their concentrations can be linked to a number of diseases, such as Parkinson’s disease and schizophrenia. Normally, in biological systems dopamine concentrations vary from about 10 nM to 1 μM, ascorbic acid from 1 to 2 mM, and uric acid from 207 to 444 μM [192]. Therefore, their accurate quantification across these concentration ranges in biological fluids is necessary for the clinical diagnosis of various diseases. As shown in Table 3, many of the CD-based systems have linear regions that extend to the lower concentration levels and while the linear regions may not extend into the higher mM levels, the biological samples can be diluted. Moreover, many of these sensors can be employed in the simultaneous detection of these analytes, with the oxidation of the molecules occurring at different potentials, enabling their resolution.

CDs have also been combined with various enzymes, antibodies, and aptamers, as illustrated in Table 4, enabling the detection of simple molecules, such as hydrogen peroxide and more complex structures. It is clearly evident that the aptamer-based sensors provide detection at very low levels. Again, rGO, MWCNTs, and metal nanoparticles, such as AuNPs, are routinely combined with the CD and enzyme to give biosensors. While MWCNTs, rGO, and metal nanoparticles feature in the fabrication of many of these sensors (Table 2, Table 3, Table 4 and Table 5), other new emerging materials, including MXenes and graphitic carbon, are now beginning to find applications in the design of CD-based sensors.

## 5. Conclusions

It is clearly evident from the papers described that CDs have excellent supramolecular recognition properties, and these can be exploited in the design and fabrication of electrochemical-based sensors for the detection of various molecules, encompassing environmental, biological, and biomedical applications. Moreover, these sensors have been employed in challenging environments. Biological and environmental media are especially difficult, with interference compounds and complex samples.

Although these CD-based electrochemical sensors have a number of attractive properties and excellent molecular recognition properties, a number of challenges still exist and must be addressed before these sensors can progress from a proof-of-concept stage to employment in environmental, biomedical, and/or healthcare sectors. While the immobilization of CDs by mixing and dispersing onto a transducer surface is a simple process and has been used widely, this method results in the gradual loss of the CD from the electrode surface. Covalent attachment of the CDs and the more recently employed approach of electropolymerization address some of these stability issues. However, more robust methods are required to give higher loadings and stronger integration of the CDs within the sensor unit.

While CDs have very good biocompatibility, many of the additives employed to enhance electron conductivity, such as rGO and CNTs, have environmental concerns, especially if they escape into the environment. In particular, carboxylated CNTs and GO, have reasonably good dispersion in water, making it more difficult to prevent their release into the environment. Therefore, these additives, which are essential in promoting efficient electron transfer, need to be effectively anchored and trapped with the CD supramolecules. In addition, the costs of CNTs remain high, and while small quantities are employed in the fabrication of sensors, this will ultimately increase the overall cost of the sensor, impacting its real applications.

Nevertheless, CDs have a promising future in the development of sensors, providing molecular recognition properties for a host of molecules, and providing a protective hydrophobic cavity for the immobilization of enzymes and aptamers. Besides, their ability to provide chiral recognition, is especially interesting in the development of chiral drug molecules. The development of new functionalized CDs with simpler synthetic steps and high yields has the potential to further extend the applications of CD supramolecules. Other new and emerging supramolecular systems, such as pillararenes, are also likely to receive more attention in the next few years, further contributing to the development of both highly sensitive and selective supramolecular electrochemical sensors. Indeed, there is potential to combine supramolecular systems such as CDs and pillararenes to give sensors that can recognize a number of analytes simultaneously.

## Figures and Tables

**Table 1:** Some examples of binding constants for the inclusion complexes formed between aromatic molecules and β-CD in the solution phase; all data taken from Liu et al. [26].

Host	log *K* (*K*/M^−1^)	−Δ*G*/kcal mol^−1^
Benzoic acid	2.74	3.73
P-nitroaniline	3.48	4.76
P-nitrophenol	2.82	3.91
L-alanine	3.37	4.60
L-aspartic acid	4.04	5.49
D-serine	2.92	3.98

**Table 2:** Summary of some of the CD-based sensors used in the determination of environmental contaminants.

Materials ^1^	Analyte	LOD	Linear Range/s	Ref.
Β-CD/AuNPs/MC	p-nitrophenol.	3.63 μg mL^−1^	0.1–10 μM 10–350 μM	[148]
α-CD/NiONPs/rGO	p-nitrophenol	0.12 nM	1–5 μM	[193]
β-CD/CB	4-nitrophenol	0.040 μM	0.125–225.8 μM	[194]
β-CD/rGO/CS	o-nitrophenol	0.018 μM	0.12–0.28 μM 5–40 μM	[69]
	p-nitrophenol	0.016 μM	0.06–0.16 μM 5–40 μM	
Β-CD/AuNP/TiO_2_NS	MP	0.05 nM	1.5–60.0 nM	[151]
β-CD/rGO	MP	0.05 ppb	0.3–1.0 ppb 1.0–500 ppb	[195]
α-CD/rGO	IDP	0.02 μM	0.5–40 μM	[21]
β-CD/MWCNTs	Bisphenol	13.76 nM	125 nM–2 μM 2–30 μM	[92]
β-CD/GO/MWCNT	Bisphenol	6 nM	0.05–5 μM	[196]
			5–30 μM	
SH-β-CD/NPGL	Bisphenol	60 nM	0.3–100 μM	[197]
β-CD-Gr/PtNPs	Bisphenol	15 nM	0.05–80 μM	[198]
β-CD/GRs/CPE	2-chlorophenol	0.2 μM	0.5–40 μM	[48]
	3-chlorophenol	0.09 μM	0.4–77 μM	
β-CD–GNs)	CBZ	2 nM	5 nM–0.45 μM	[45]
β-CD/rGO/PU	TER	0.550 μM	2.50–30 μM	[199]
	NIM	0.083 μM	0.62–7.3 μM	
	MET	0.077 μM	0.62–7.3 μM	
β-CD/MGO	TC	0.18 ng L^−1^	0.5–90.0 ng L^−1^	[47]
	DC	0.18 ng L^−1^	0.5–90.0 ng L^−1^	
β-CD/PdNPs/rGO	Hydrazine	28 nM	0.05–1600 μM	[200]
β-CD/PANI/MWCNT	MCPA	1.1 μM	10–50 μM	[102]
	Chloromethylphenol	1.9 μM	10–50 μM	
β-CD/MWCNT	MCPA	0.99 μM	10–100 μM	[101]
Bi/HP-β-CD-rGO/Nafion	Pb(II)	0.09 nM	1.0–90 nM	[201]
(stripping voltammetry)	Cd(II)	0.07 nM	5.0–90 nM	
β-CD/NH_2_-rGO/	Cu(II)	2.8 nM	0.05–1.0 μM	[202]
mp-g-C_3_N_4_/β-CD	TNT	68 ppb	1–100 μM	[203]
CNHs/β-CD	CNB	9.0 nM	0.05–1.0 μM	[153]
MXene/CNHs/βCD/MOF	CBZ	1.0 nM	3.0 nM–10.0 μM	[154]

^1^ Abbreviations: limit of detection (LOD); 2,4,6–trinitrotoluene (TNT); 1–chloro–4–nitrobenzene (CNB); carbon nanohorns (CNHs); terbutaline (TER); nimesulide (NIM); methocarbamol (MET); polyurethane (PU); methyl parathion (MP); mesoporous carbon (MC); carbon black (CB); nanosheets (NS), chitosan (CS); imidacloprid (IDP); carbendazim (CBZ); gold leaf (NPGL); tetracycline (TC); doxycycline (DC); magnetic GO (MGO); carbon paste electrode (CPE).

**Table 3:** Summary of some of the CD-based sensors used in the electrochemical detection of essential biomolecules and amino acids.

Materials ^2^	Analyte	LOD	Linear Range/s	Ref.
β-CD/rGO	Ascorbic acid	0.067 mM	0.2–2 mM	[129]
	Dopamine	0.017 μM	0.05–50 μM	
	Uric acid	0.026 μM	0.08–150 μM	
3DrGO/Fe_3_O_4_/HP-β-CD	Serotonin	3.3 nM	0.01–25 μM	[204]
	Dopamine	6.7 nM	0.02–25 μM	
	Ascorbic acid	3.3 μM	10–350 μM	
β-CD/CQDs	Ascorbic acid	0.14 μM	4–220 μM	[205]
	Uric acid	0.01 μM	0.3–200 μM	
	Trypophan	0.16 μM	5–270 μM	
Au/NF/β-CD/AuNPs	Dopamine	0.6 nM	0.05–280 μM	[159]
MWCNT/β-CD	Dopamine	6.7 μM	0.01–0.08 mM	[206]
GR/CD/Pt	Cysteine	0.12 μM	0.5–170 μM	[207]
(CuO/β-CD/NF	Tyrosine	0.0082 μM	0.01–100 μM	[208]
PDA-β-CD	Glycine	0.2 μM	0.20–70 μM	[209]
	Cysteine	0.06 μM	0.06–0.2 μM	
	Tyrosine	0.01 μM	0.01–0.1 μM	
	Phenylalanine	0.2 μM	0.20–10 μM	

^2^ Abbreviations: carbon quantum dots (CQDs); Nafion (NF); polydopamine (PDA).

**Table 4:** A selection of some of the biosensors using CDs or functionalized CDs as a supramolecular recognition unit.

Enzyme/Protein/Other Reagents ^3^	Target Analyte	Linear Range	LOD	Ref.
HRP	H_2_O_2_	100 mM–3.9 mM	2 μM	[166]
HRP/BSA/MB	H_2_O_2_	–	0.5 μM	[210]
GOX/CNT	Glucose	4 mM–3.2 mM	3.5 μM	[211]
CD/rGO/Cu@Ag-CD/antibodies	CEA	0.01–20 ng mL^−1^	20 fg mL^−1^	[168]
Fe_3_O_4_/TYR	Cathecol	100 nM–12 μM	22 nM	[161]
Fe_3_O_4_/XO	Xanthine	5.0–120 μM	2.0 μM	
XO	Xanthine	300 μM–10.4 mM	200 μM	[162]
SPE/rGO	Eugonal	13 nM–10 μM	4 nM	[212]
Lac/rGO	Dopamine	0.1–70 μM	30 nM	[213]
CD/rGO/AuNPs/PB/CS	Malathion Carbaryl	7.98−2.00 × 10^3^ pg mL^−1^ 4.3−1.00 × 10^3^ pg mL^−1^	4.14 pg mL^−1^ 1.15 pg mL^−1^	[66]
Aptamer/AuNPs/GO/ferrocene	Thrombin	1.6 × 10^–17^–8.0 × 10^–15^ M	5.2 × 10^–18^ M	[178]
Aptamer/ferrocene	Tetracycline	0.01–100 nM	0.008 nM	[179]

^3^ Abbreviations: xanthine oxidase (XO); soybean peroxidase (SPE); laccase (Lac) tyrosinase, (TYR); Prussian blue (PB); chitosan (CS).

**Table 5:** Summary of some of the CD-based sensors employed in the electrochemical determination of drugs and flavonoids.

Materials ^4^	Analyte	LOD	Linear Range/s	Ref.
β-CD/AgNPs/GO/G-DVD	Naproxen	0.08 μM	0.4 μM–80 μM	[214]
β-CD-l-arg	Ciprofloxacin	0.01 μM	0.05–100 μM	[215]
	Ofloxacin,	0.04 μM	0.1–100 μM	
	Norfloxacin	0.04 μM	0.1–40 μM	
	Gatifloxacin	0.02 μM	0.06–100 μM	
DM-β-CD-GNs	Isoquercitrin	4 nM	10 nM–3.0 μM	[56]
	Baicalin	10 nM	0.04 μM–3.0 μM	
Poly-βCD/rGO	Quercetin	0.001 µM	0.005–20 µM	[216]
β-CD/CB	Flutamide	0.016 µM	0.05–158 µM	[194]
SH-β-CD-Gr/PdNPs	Rutin	0.3 nM	1.0 nM–30 µM	[58]
	Isoquercitrin	1.6 pM	5.0 pM–5 µM	
PDDA-G-CNTs/β-CD	Luteolin	0.02 μM	0.05–60 μM	[217]
β-CD-AuNP/GO	Nilutamide	0.4 nM	0.01–193 μM	[218]
β-CD/CNF	Nitrofurantoin	1.8 nM	0.004–308 µM	[219]
β-CD/ILC/rGO	Methocarbamol	6.64 pM 10.9 nM	0.04–1 µM 8–100 µM	[190]
GR-β-CD/CuONPs	metronidazole	0.6 nM	0.002–210.0 µM	[220]
SnS_2_/GO/β-CD	Melatonin	0.17 nM	1 nM–100 μM	[221]
β-CD/rGO	Acetaminophen	2.3 μM	0.01–0.8 mM	[44]
SnO_2_/MWCNT/β-CD	Acetaminophen	5.8 nM	0.01–340 μM	[222]
PB-rGO/Tβ-CD/AuNPs	Quercetin	1.83 nM	0.005–0.4 μM	[57]
NH_2_-GQDs/Au-β-CD	Quercetin	285 pM	1–210 nM	[61]
DM-β-CD-GNs	Isoquercitrin	4 nM	10 nM–3.0 μM	[56]
	Baicalin	10 nM	0.04–3.0 μM	
MIP/CoNPS/β-CD/MWNTs	Oxacillin	6.9 nM	0.2–100 μM	[89]

^4^ Abbreviations: gold digital versatile disc (G–DVD); l-arginine (l-arg); poly(diallyldimethylammonium chloride) (PDDA); carbon nanofiber (CNF); ionic liquid crystal (ILC); 1-pyrenebutyrate (PB); mercapto–β-cyclodextrin (Tβ-CD); aminated graphene quantum dots (NH_2_–GQDs); 2,6–dimethyl–β-cyclodextrin (DM–β-CD); molecularly imprinted polymers (MIP).

## Data Availability

Data sharing not applicable.
